# Genome Sequence of an Australian Monophasic *Salmonella enterica* subsp. *enterica* Typhimurium Isolate (TW-Stm6) Carrying a Large Plasmid with Multiple Antimicrobial Resistance Genes

**DOI:** 10.1128/genomeA.00793-17

**Published:** 2017-08-17

**Authors:** Mike L. Dyall-Smith, Yuhong Liu, Helen Billman-Jacobe

**Affiliations:** a Veterinary Biosciences, Faculty of Veterinary and Agricultural Sciences, University of Melbourne, Parkville, Victoria, Australia; b Asia-Pacific Centre for Animal Health, Faculty of Veterinary and Agricultural Sciences, University of Melbourne, Parkville, Victoria, Australia; c National Centre for Antimicrobial Stewardship, Peter Doherty Institute, Parkville, Victoria, Australia

## Abstract

We report the genome sequence of a monophasic *Salmonella enterica* subsp*. enterica* Typhimurium strain (TW-Stm6) isolated in Australia that is similar to epidemic multidrug-resistant strains from Europe and elsewhere. This strain carries additional antibiotic and heavy-metal resistance genes on a large (275-kb) IncHI2 plasmid.

## GENOME ANNOUNCEMENT

Monophasic variants of *Salmonella enterica* subsp*. enterica* Typhimurium with the antigenic formula 1,4,[5],12:i− are commonly associated with pigs, cattle, and humans. Several clones which vary in the number and arrangement of antimicrobial resistance (AMR) genes have independently emerged and spread globally (1). The recently described European clade carries at least *strA*, *strB*, *sul2*, *tetB*, and mercury resistance genes on a locus inserted into the *fljAB* chromosomal region, and copper resistance genes on a novel genomic island SGI-3 (2). We describe a monophasic *S*. Typhimurium strain isolated in Australia from pig feces wherein the chromosome resembles the European clade; however, it also harbors a large conjugative plasmid carrying AMR genes and additional heavy-metal resistance genes.

Genomic DNA was extracted from pure isolates using the JANUS Chemagic automated workstation (PerkinElmer) with the Chemagic Viral DNA/RNA kit (PerkinElmer). Unique dual-indexed libraries were prepared using the Nextera XT DNA sample preparation kit (Illumina) and sequenced on the Illumina NextSeq 500 with 150-cycle paired-end chemistry (80-fold coverage) as described by the manufacturer’s protocols. Long-read sequencing was performed on the Pacific Biosciences (PacBio) RS II platform. A 10-kb to 20-kb library was prepared and sequenced (28-fold coverage) using C4/P6 chemistry on single-molecule real-time cells on the PacBio RS II. A hybrid assembly of paired-end Illumina short and PacBio long reads was *de novo* assembled using Unicycler, and the assembly was polished with Pilon (3, 4). A single chromosomal contig of 4,999,862 bp was generated with a G+C content of 52.2%. Automated annotation via the NCBI Prokaryotic Genome Annotation Pipeline was refined manually using PATRIC (5) and EcoGene 2.0, within the Geneious v10 environment (6). A 275,801-bp IncHI2 resistance plasmid, pSTM6-275, and a 4,083-bp, cryptic, MOBQ plasmid (4 open reading frames [ORFs]), pSTM6-4, were also present. Plasmid pSTM6-275 carried 290 ORFs including genes for AMR, copper resistance, and HipA/HipB toxin/antitoxin genes.

The chromosome of *S*. Typhimurium TW-Stm6 had 4,808 annotated ORFs and showed a 15,725-bp deletion of the *fliAB* region (relative to *S*. Typhimurium SL1344) with a concomitant 28,209-bp insertion of a sequence containing multiple AMR and metal resistance genes, arranged in a manner similar to that of the European strain SO4698-09 (2). Complete IS elements or transposons (23 on the chromosome, 17 on pSTM-275) were present, particularly IS26 and IS200, as well as numerous intergenic repeats (359 REP [7]; 34 ERIC [8]), including 19 RSA-like repeats (9) that we have designated SPED repeats for *Salmonella* palindromic extragenic dispersed repeats.

### Accession number(s).

This complete genome project has been deposited in DDBJ/ENA/GenBank under accession no. CP019647, CP019648, and CP019649.
